# Association of Genetic Polymorphisms in *GSTP1, GSTM1*, and *GSTT1* Genes with Vesicoureteral Reflux Susceptibility in the Children of Southeast Iran

**DOI:** 10.18502/ijph.v49i7.3591

**Published:** 2020-07

**Authors:** Sima SHAHROKHZADEH, Azam SOLEIMANI, Dor-Mohammad KORDI-TAMANDANI, Mohammad Hossein SANGTARASH, Omid NEJATI, Mohsen TAHERI

**Affiliations:** 1. Department of Biology, University of Sistan and Baluchestan, Zahedan, Iran; 2. Legal Medicine Research Center, Legal Medicine Organization, Tehran, Iran; 3. Department of Paramedics, Mashhad Medical Sciences Branch, Islamic Azad University, Mashhad, Iran; 4. Genetics of Non-Communicable Diseases Research Center, Zahedan University of Medical Sciences, Zahedan, Iran

**Keywords:** Vesicoureteral reflux, Genetic susceptibility, Glutathione S-transferase, Genetic polymorphisms

## Abstract

**Background:**

Vesicoureteral reflux (VUR) disease is the most common type of urinary tract anomalies in children. Genetic risk factors may be associated with the etiology of VUR. The role of the Glutathione S-transferases (*GSTs*) as multifunctional enzymes is cellular oxidative stress handling. This is the first study aimed at evaluating the relative risk of *GSTP1, GSTM1,* and *GSTT1* polymorphisms in VUR susceptibility in children and provides new important insights into the genetics of affected children.

**Methods:**

The study was done in 2013 in Sistan and Baluchestan University, eastern Iran. Genotyping of three *GSTP1, GSTM1,* and *GSTT1* genes were determined using the multiplex polymerase chain reaction assay in 216 reactions for 72 VUR children and 312 reactions for 104 healthy controls.

**Results:**

The presence of *GSTT1* deletion was associated with high risk of VUR in children, whereas *GSTP1* and *GSTM1* genotypes did not show the same effect. Furthermore, the combination of *GSTT1/GSTM1* and *GSTT1/ GSTP1* genotypes showed a significant influence on lower risk of VUR in children.

**Conclusion:**

Deletion of *GSTT1* functional gene is a genetic risk factor causing VUR in children. Interestingly, the combination of *GSTM1* and *GSTP1* null genotypes with *GSTT1* has shown a protective role against risk of *GSTT1* deletion.

## Introduction

*Vesicoureteral reflux* **( ***VUR***)** disease is an abnormal condition in which urine retrogrades from the bladder into the ureters and kidneys (1). It is the most common congenital urological anomaly in children and may be observed in two forms like primary and secondary (1). The primary VUR disease has been reported in 1%–2% of the pediatric population and 30%–40% of children with urinary tract infections (1–5). The secondary condition is due to high blood pressure factors in the bladder, such as neurogenic bladder and obstructive factors (1). The outbreak of VUR in 27%–51% of siblings and 66% of off springs of known VUR patients suggests that VUR is often hereditary (6–9). VUR is considered to be a complex disease with different patterns of inheritance such as autosomal dominant with incomplete penetrance (10, 11), autosomal recessive (12) X-linked (13), and polygenic (14).

In the pathogenesis of VUR, several genes play a pivotal role (15). Here, we will focus our attention on *GST*s gene polymorphisms. *GST*s are members of a multigene family of metabolic enzymes divided into four major subfamilies designated as *GST*α (*GSTA1*), *GSTμ (GSTM1), GSTθ* (*GSTT1*) and *GST* π (*GSTP1*). These enzymes as cell housekeepers protect cells against electrophiles and oxidative stressors in the environment by detoxifying a wide variety of potentially toxic and carcinogenic electrophiles (16,17). *GSTP1, GSTM1* and *GSTT1* genes are located on chromosomes 11q13, 1p13.3 and 22q11.2 respectively (18). In the *GSTP1*, exon 5 is rs1695 polymorphism with an A→G transition at nucleotide 313, leads to replacement of valine for isoleucine (19). The *GSTM1* and *GSTT1* null genotypes are referred to as deletions in the sequence of these genes that caused by homologous recombination of a number of repeats spanning around them (20). It was identified that detoxification effects modified by *GST*s polymorphism possibly can aggravate the susceptibility to diseases (18,21). The magnitude of the influences of *GST* genes polymorphism distribution on various diseases has been extensively studied (22–24).

Our goal was to assess the influence of *GST* genes polymorphism on VUR susceptibility in the Iranian children.

## Material and Methods

### Subjects

In February, 2013, a case control study was conducted on 176 samples, including 72 children with VUR disease diagnosed at different stages of disease progression and 104 healthy children as a control group. Three *GSTP1, GSTM1,* and *GSTT1* polymorphisms were evaluated in patients and healthy subjects. 216 reactions were done for patients and 312 for healthy subjects. The group of control were children who did not have any history of VUR and urinary tract diseases.

The current study was a student thesis. The parents of the children entered the study with informed consent and voluntary participation of the children. The study was approved by the Ethics Committee of the University of Sistan and Baluchestan as per proposal and protocol of study code 2011.7170.

### Genomic DNA Extraction and PCR Mix Preparation

DNA from the whole blood was extracted by the salting-out method described by Miller et al (25). Concentration and purity of DNA were determined by DNA electrophoresis and spectrophotometer. Primers used in reactions of PCR were selected according to the previous study (26) then verified using database of single nucleotide polymorphisms (SNPs) (dbSNP 129; https://www.ncbi.nlm.nih.gov/projects/SNP/) and BLAST website (http://blast.ncbi.nlm.nih.gov/Blast.cgi).

The PCR mixtures of *GSTM1*, *GSTT1* and, *GSTP1* genes were prepared in volumes of 20 μl containing 10 μl master mix PCR (Parstous 1x.Iran), 100 ng genomic DNA, and 1 μl (10 pM) of each forward and reverse primers.

### GSTP1 Polymorphism

Tetra primer amplification refractory mutation system–polymerase chain reaction (T-ARMS-PCR) used for amplifying the region that comprises of 467bp fragment of the *GSTP1* gene polymorphism with two non-allele-specific primers as the outer primers (Table 1). Two bands of 233 bp and 290 bp were observed with two allele-specific primers as the inner primers (Table 1). PCR was performed in a total volume of 20 μl containing 10 μl master mix PCR (Parstous 1x.Iran), 100 ng genomic DNA, 1 μl (10 pM) of each primers (inner and outer primers). PCR program started at 94 °C initial denaturation temperature for 5 min followed by 40 cycles at 95 °C denaturation temperature for 40 sec, 60 °C annealing temperature for 30 sec, 72 °C extension temperature for 30 sec, and 72 °C as final extension temperature for 10 min. Finally, amplification products separated by loading in 2% aga-rose gel electrophoresis stained by green viewer.

**Table 1:** The Features of Primers used to Amplify the *GSTP1*, *GSTT1*, and *GSTM1* Genes Polymorphism

*** Genes ***	*** Sequence ***	*** Fragment length (bp) ***
* GSTP1 - exF *	5′-CAGGTGTCAGGTGAGCTCTGAGCACC-3′	467
* GSTP1 - exR *	5′-ATAAGGGTGCAGGTTGTGTCTTGTCCCA-3′	
* GSTP1 -inF *	5′-CGTGGAGGACCTCCGCTGCAAATCCA-3′	233 A allele
* GSTP1 * -inR	5′-CTCACATAGTTGGTGTAGATGAGGGATAC-3′	290 G allele
* GSTT1 * -F	5′- TTCTGCTTTATGGTGGGGTC-3′	542
* GSTT1 * -R	5′- GTGATGTTCCCTGTTTTCCT-3′	
* GSTM1 * -F	5′-GCTGCCCTACTTGATTGATG-3′	325
* GSTM1-R *	5′-CCCCAAATCCAAACTCTGTC-3′	

### GSTT1 and GSTM1 Polymorphisms

In cases with no deletion in *GSTM1* and *GSTT1* genes polymorphism, PCR products with 542bp and 325bp bands were assigned as *GSTM1* and *GSTT1* (present allele) respectively by a conventional PCR reaction. However, in cases with deletion, amplification was not performed thus no band was observed (homozygous null genotypes). The PCR program of *GSTT1* gene was set at 94 °C for 5 min and then followed by 35 cycles at 94 °C as initial denaturation temperature for 60 sec, 59 °C annealing temperature for 60 sec, 72 °C extension temperatures for 40 sec, and the *GSTM1* gene was set at 94 °C for 5 min and then followed by 25 cycles at 95 °C as initial denaturation temperature for 20 sec, 60 °C annealing temperature for 30 sec, 72 °C extension temperatures for 30 sec, and 72 °C final extension temperature for 10 min. Finally, amplification products loaded in 2% agarose gels stained by green viewer.

### Statistical Methods

The collected data from *GSTP1*, *GSTT1* and *GSTM1* genotypes were processed with the statistical analysis software SPSS (ver.16, Chicago, IL, USA). Distribution of allele frequencies and genotypes of *GSTP*, *GSTT1* and *GSTM1* were estimated by Chi-square and Fischer’s exact t-test in children with VUR and healthy controls. Moreover, statistical comparisons were calculated with Odds ratio (OR) and 95% confidence intervals (CIs) between two groups. The p values was ≥ 0.05 regarded as statistically significant.

## Results

### Subject’s Data and Genotyping

Average age and weight of all subjects with VUR were 2.51 ± 2.89 yr and 11.56 ± 6 kg, respectively. The average age and weight of selected healthy children were 2.79 ± 5.9 year and 11.23 ± 5.9 kg. Distribution frequency of polymorphisms studied were in Hardy-Weinberg equilibrium. The analysis has been conducted according to the most frequent genotypes as a reference. The desired fragments of PCR products for *GSTP1*, *GSTT1* and *GSTM1* genotypes were revealed after electrophoresis.

### GSTP1 Gene Polymorphism and VUR Patient Risk in Children

To analyze the *GSTP1*, the AA genotype was considered as reference. When the other two genotypes AG and GG were compared with the reference genotype, it appeared that there was not a significant difference for *GSTP1* gene between A and G allele frequencies in the children with VUR and healthy group (Table 2).

**Table 2:** *GSTP1* Gene Genotypes Frequency in Children with VUR Disease(72) and Control Group(104)

*** GSTP1 ***	*** Cases, n(%) ***	*** Controls, n(%) ***	*** OR ***	*** CI(95%) ***	*** P value ***
AA genotype	26(36.11)	37(35.57)	-	-	Ref
AG genotype	41(56.94)	60(57.69)	0.97	0.49 – 1.94	1.00
GG genotype	5(6.94)	7(6.73)	1.02	0.23 – 4.20	1.00
AG+GG genotypes	46(63.88)	67(64.42)	0.98	0.50 – 1.92	1.00
A Allele	93(0.65)	134(0.64)	-	-	Ref
G Allele	51(0.35)	74(0.36)	1	0.64-1.57	1.00

### GSTT1 Gene Polymorphism and VUR disease risk in children

As seen in Table 3, a statistically significant difference was found between the deletion of *GSTT1* gene polymorphism in children affected by VUR disease and healthy children group (*P-*value=0.004). With odds ratio higher than one, a correlation was found between the *GSTT1* Null genotype and increased risk of VUR disease occurrence in children (OR 3.14, CI 1.4387 – 6.8745) (Table 3).

**Table 3:** Frequency of *GSTT1* and *GSTM1* Genes Genotypes in Children with VUR Disease and Control Group

*** GENES ***	*** Alleles ***	*** Case, n(%) ***	*** Controls, n(%) ***	*** OR (CI(95%)) ***	*** P-value ***
* GSTT1 *	Present	62(86.11)	69(66.35)	-	Ref
	Null	10(13.89)	35(33.65)	3.14(1.43–6.87)	0.004
* GSTM1 *	Present	35(48.61)	53(50.96)	-	Ref
	Null	37(51.39)	51(49.4)	1.09(0.60–2.00)	0.887

Present=Non Deletion, Null=Deletion/ Homozygous Null Genotypes

### GSTM1 Gene Polymorphism and VUR Risk in Children

No statistically significant correlation was found in children affected by VUR *compared* to *controls*, when null allele genotype was considered as reference (Table 3).

### GSTM1 and GSTT1 Combined Genotypes in Children with VUR Disease

The combination of *GSTM1* and *GSTT1* genotypes showed a significant correlation with lower risk of VUR when it compared to *GSTM1* present/*GSTT1* present genotype (*P*-value=0.023, OR= 0.25, CI 0.06–0.89) (Table 4).

**Table 4:** Combination of Genotypes of GSTP1 and GSTM1/GSTT1 Polymorphisms and Vesicoureteral Reflux Susceptibility in Children with VUR Disease and Control Group

*** GSTs genotypes ***	*** Cases, n(%) ***	*** Controls, n(%) ***	*** OR ***	*** CI(95%) ***	*** P value ***
AA * GSTP1 * /Present * GSTM1 *	12(16.66)	19(18.27)	-	-	Ref
AG * GSTP1 * /Present * GSTM1 *	18(25)	29(27.88)	0.98	0.35 – 2.79	1.00
GG * GSTP1 * /Present * GSTM * 1	5(6.94)	5(4.81)	1.57	0.29 – 8.47	0.71
AA * GSTP1 * / Null * GSTM * 1	14(19.44)	18(17.31)	1.23	0.40 – 3.80	0.80
AG * GSTP1 * / Null * GSTM * 1	23(31.94)	31(29.81)	1.17	0.44 – 3.22	0.82
GG * GSTP1 * / Null * GSTM * 1	0(0)	2(1.92)	0.00	0.00 – 9.39	0.52
AA * GSTP1 * / Present * GSTT * 1	20(27.78)	20(19.23)	-	-	Ref
AG * GSTP1 * / Present * GSTT1 *	37(51.39)	45(43.27)	0.82	0.36 – 1.88	0.70
GG * GSTP1 * / Present * GSTT * 1	5(6.94)	4(3.85)	1.24	0.23 – 7.25	1.00
AA * GSTP1 * /Null * GSTT * 1	6(8.33)	17(16.35)	0.36	0.10 – 1.21	0.11
AG * GSTP1 * / Null * GSTT * 1	4(5.56)	15(14.42)	0.27	0.056 – 1.06	0.048
GG * GSTP1 * / Null * GSTT * 1	0(0)	3(2.88)	0.00	0.00 – 2.72	0.24
* GSTT1 * Present /Present	31(43.06)	35(33.65)	-	-	Ref
* GSTM1 *					
* GSTT1 * Present * / * Null * GSTM1 *	31(43.06)	34(32.69)	1.03	0.49 – 2.16	1.00
* GSTT1 * Null * / * Present * GSTM1 *	4(5.55)	18(17.31)	0.25	0.06 – 0.89	0.023
* GSTT1 * Null * / * Null * GSTM1 *	6(8.33)	17(16.35)	0.40	0.12 – 1.24	0.092

Present=Non Deletion, Null=Deletion/ Homozygous Null Genotype

### GSTM1 and GSTP1 Combined Genotypes and VUR Disease in Children

*GSTT1* and *GSTP1* combined genotypes did not reveal a meaningful relationship with risk of VUR in children (Table 4).

### GSTT1 and GSTP1 Combined Genotypes and VUR Disease in Children

*GSTP1* AG/*GSTT1* null combined genotypes compared to *GSTP1* AA/*GSTT1* present genotypes showed a significant correlation with lower risk of VUR in children (*P*-value=0.048, OR 0.00, CI 0.00–2.72) (Table 4).

## Discussion

In this study, **t**he *GSTT1* gene deletion in children with the VUR disease is significantly higher than those in control group. Therefore, there is an increased risk of VUR disease in children with *GSTT1* null genotype. Other studies have shown the deletion of *GSTT1* gene does have a close association with the Brazilian acute promyelocytic leukemia and psoriasis in North India.

In addition, the *GSTM1/GSTT1* null genotypes are related to increased susceptibility to acute promyelocytic leukemia (27,28).

In this study, the *GSTP1* and *GSTM1* genotypes does not indicate a significant risk of increased susceptibility to VUR disease. But there is a significant correlation between reduced risk of the VUR disease and a combination of *GSTM1* present and *GSTT1* null polymorphism. Furthermore, combination of *GSTP1* AG/*GSTT1* null significantly reduces the risk of VUR disease in children. *GSTM1* present and *GSTP1* AG geno-types have a very strong compensating effect on the deletion of *GSTT1* gene.

There are other researches on *GSTs* genes. For example, in one study, *GSTM1*-null and *GSTP1* Val allele genotypes, were found to increase the risk of nonalcoholic fatty liver in the Iranian population (26). A significant association was found between *GSTM1* null genotype and *GSTT1* gene polymorphism in inflammatory bowel diseases (29). Another study discovered *GSTM1* and *GSTT1* null genotype are associated with male infertility (30). A research result reported a significant relationship between *GSTT1* null polymorphism and chronic myeloid leukemia (31). By contrast, *GSTP1* Val is associated with the decreased risk of premalignant lesions in another study (32). A combination of *GSTM1* present and *GSTT1* null genotypes have a protective role against susceptibility to chronic myeloid leukemia (31). *GSTP1*Val allele reduces the risk for premalignant and endoscopic gastric lesions, whereas *GSTM1* and *GSTT1* null genotypes increases it. (32). *GSTM1* and *GSTT1* null polymorphisms are associated with risk factors causing the Asian breast cancer and also *GSTP1* Val105Ile (rs1695) polymorphism is a risk factor for Caucasians breast cancer (33).

Carriers of *GSTP1, GSTM1* and *GSTT1* polymorphisms tend to show a supportive effect for detoxification activity of *GST*. Thus, these polymorphisms activate a defense response against toxic metabolites. Deleting genes of *GST* could decrease detoxification of harmful electrophiles associated with *GST* activity and DNA stability, which results in susceptibility to various diseases (22–24). There are contradictory results in the studies on the effect of *GSTs* polymorphism on Atherosclerosis due to demographic diversity of the studied population (34). *GSTs* cellular detoxification activities involve in inflammatory processes, cellular differentiation and signaling pathways (35–37). Studies performed in past years have revealed that oxidative stress worsens diseases caused by inflammatory response (38,39). Studies demonstrated *GSTs* enzyme activities always depend on their genotype. Therefore, a specific genotype of *GST* genes can lead to reduced enzymatic activity (40,41).

The goal of VUR treatment is to reduce urinary tract infection, inflammation, kidney scars control and other complications caused by this abnormality in children. Combination of various deletions lead to pharmacology, toxicology and hereditary differences which theoretically increases the risk of various diseases (42,43). Thus, *GST*s genes may prove effective in managing VUR infection and scar prevention.

## Conclusion

This study suggests a correlation between deletion of *GSTT1* gene and increased risk of VUR disease in children. However, *GSTP1* Ile/Val and *GSTM1* act in a preventative role against susceptibility to VUR disease, given deletion of *GSTT1* gene.

## Ethical considerations

Ethical issues (Including plagiarism, informed consent, misconduct, data fabrication and/or falsification, double publication and/or submission, redundancy, etc.) have been completely observed by the authors.
